# Lived religion and gendered environmental governance: ecofeminism and indigenous cosmology in the Kajang community, Indonesia

**DOI:** 10.3389/fsoc.2026.1902355

**Published:** 2026-07-28

**Authors:** Najamuddin Najamuddin, Fadhillah Sri Meutia, Sopian Tamrin, Abdul Rahman, Arisnawawi Arisnawawi

**Affiliations:** 1 Department of Sociology - Anthropology, Faculty of Social Sciences and Law, Universitas Negeri Makassar, Makassar, Indonesia; 2 Department of Political Science, Faculty of Social and Political Sciences, Universitas Negeri Surabaya, Surabaya, Indonesia

**Keywords:** ecofeminism, environmental governance, indigenous cosmology, lived religion, religious authority, sacred ecology, sustainability

## Abstract

This article examines how lived religion, indigenous cosmology, and gendered moral practice shape environmental governance within the Kajang indigenous community of South Sulawesi, Indonesia. Drawing on qualitative fieldwork with 27 participants and secondary ecological data, the study demonstrates that ecological sustainability is sustained not through formal institutional regulation but through culturally embedded systems of lived religion and collective moral obligation. Central to this system is the cosmological concept of anrongta, which positions nature as a sacred maternal entity requiring protection, restraint, and reciprocal ethical responsibility. This cosmology is operationalized through a structured forest zoning system (Borong Karama, Borong Batasayya, Borong Luarayya) reinforced by customary sanctions (ba’bala) and ritual practices. The findings further reveal that women occupy central positions through the institutionalized role of Anrongta, functioning as advisor to the Ammatoa and participant in customary deliberations. Significantly, the Kajang case reveals a complex gender dynamic in which feminist goals of environmental care are enforced through masculine mechanisms, demonstrating that ecofeminism in this context transcends essentialist binaries. By integrating lived religion, ecofeminism, and indigenous cosmology, this article argues that cosmologically embedded governance constitutes a form of sacred ecology in which religion, gender, morality, and environmental sustainability are mutually embedded within ordinary social life. The study contributes to the sociology of religion by expanding understandings of lived religion beyond institutional settings and by demonstrating how indigenous religious systems may function as culturally grounded mechanisms of sustainability governance.

## 1 Introduction

In recent decades, escalating ecological crises including climate change, biodiversity loss, deforestation, and environmental degradation have intensified scholarly interest in the relationship between religion and environmental sustainability. Earlier scholarship frequently associated religion, particularly monotheistic traditions, with anthropocentric worldviews that legitimized human domination over nature (White, 1967). However, subsequent studies have increasingly reconsidered religion not only as a system of belief but also as a source of ecological ethics, moral obligation, and environmental responsibility (Grim, 2001; Grim and Tucker, 2014; Taylor, 2009). This shift has encouraged scholars to examine how religious systems shape environmental behavior, collective responsibility, and sustainability practices across diverse social and cultural contexts.

At the same time, critical environmental scholarship increasingly argues that contemporary ecological crises cannot be adequately addressed through technocratic regulation and state-centered environmental policy alone (Dryzek, 2021; Escobar, 2018; Galiñanes and Klinkers, 2026). Sacred ecological governance also depends upon culturally embedded systems of meaning, moral discipline, collective participation, and socially grounded understandings of human–nature relations. Consequently, scholars have increasingly emphasized the importance of indigenous ecological knowledge, sacred ecology, and community-based environmental practices in sustaining ecological continuity and collective environmental responsibility (Berkes, 2017; Ingold, 2021; Kohn, 2013).

Within the sociology of religion, recent developments in lived religion scholarship further contribute to these discussions by emphasizing religion as embodied social practice rather than solely institutional doctrine or formal theology (Ammerman, 2021; McGuire, 2008; Orsi, 2016). From this perspective, religion operates through ordinary routines, material interaction, embodied discipline, and everyday moral conduct. Religious meaning is therefore reproduced not only through formal institutions but also through practical activities shaping social relations, moral obligation, and ecological behavior within everyday life.

This perspective is particularly important for understanding indigenous societies in which religion, cosmology, morality, and environmental relations are deeply interconnected. In many indigenous communities, forests, rivers, mountains, and ecological spaces are not understood merely as material resources but as sacred entities embedded within broader cosmological systems linking humans, ancestors, spirits, and the natural world (Descola, 2013; Ingold, 2021). Environmental preservation therefore emerges through ritual participation, customary norms, collective discipline, and moral responsibility rather than through external environmental enforcement alone.

At the same time, gender remains a crucial yet comparatively underexplored dimension within scholarship on religion and ecology. Ecofeminist scholarship has long emphasized the relationship between women and environmental sustainability, particularly in contexts where ecological care, subsistence, and moral responsibility are socially gendered (Agarwal, 2010; Plumwood, 1993; Shiva, 1988). More recent scholarship has expanded these discussions by emphasizing intersectionality, cultural specificity, and locally situated environmental practices (Arora-Jonsson, 2011). Nevertheless, much ecofeminist scholarship continues to be shaped primarily by secular and Western theoretical frameworks, resulting in relatively limited engagement with indigenous cosmologies and lived religious environmental practices within non-Western societies.

Despite the growing literature on religion and sustainability, important gaps remain. Existing studies frequently focus on institutional religion, environmental ethics, or policy-oriented approaches while giving comparatively limited attention to everyday religious practice and indigenous cosmological systems shaping ecological conduct. Similarly, studies of indigenous environmental governance often treat religion as background culture rather than as an active moral and social force organizing ecological behavior. At the same time, women’s environmental participation is frequently analyzed primarily as labor or caregiving activity rather than as a form of socially embedded religious authority reproduced through ordinary practice and ritual participation.

This article addresses these gaps by examining how lived religion, indigenous cosmology, and gendered ecological participation intersect in shaping cosmologically embedded governance within the Kajang indigenous community of South Sulawesi, Indonesia. The Kajang community represents an important case because of its continuing commitment to Pasang ri Kajang, a customary moral system regulating ritual life, social conduct, and ecological relations. Within Kajang cosmology, the forest is understood through the concept of anrongta nature as a nurturing mother requiring protection, restraint, and reciprocal moral responsibility. Environmental preservation therefore functions not merely as practical conservation but as a sacred moral obligation embedded within collective social life.

Importantly, women occupy central positions within this religious-ecological system through ritual participation, domestic ecological regulation, intergenerational moral transmission, and the maintenance of customary values. Their authority emerges less through formal institutional leadership than through everyday religious practice and socially embedded ecological stewardship. The Kajang case therefore provides an important opportunity to examine how environmental sustainability may be reproduced through decentralized forms of religious authority operating within ordinary communal life.

This study is guided by three central questions. First, how does Kajang cosmology construct environmental preservation as a form of lived religious practice? Second, in what ways do women enact religious authority through ecological stewardship and ritual participation? Third, how does sacred ecology function as a culturally embedded system of environmental governance within everyday Kajang social life?

To address these questions, this article develops an integrated analytical framework combining lived religion, ecofeminism, and indigenous cosmology. This framework enables analysis of how ecological sustainability is reproduced through ritual participation, embodied moral discipline, cosmological belief, and gendered social responsibility operating within everyday community life. Rather than treating religion, gender, and ecology as separate analytical domains, this study conceptualizes them as mutually embedded dimensions of socially organized moral ecological governance.

The central argument advanced in this article is that the religiously embedded environmental system within the Kajang community operates through a form of sacred ecology in which cosmological belief, lived religious practice, gendered moral participation, and ecological discipline are mutually reinforcing. Within this system, women function as important religious-ecological actors whose authority is reproduced through everyday practice, ritual continuity, and intergenerational moral transmission rather than formal institutional leadership alone.

This study contributes to the sociology of religion in several important ways. First, it extends the concept of lived religion by demonstrating how ecological practices themselves may function as forms of religious action embedded within ordinary social life. Second, it expands ecofeminist scholarship by showing how women’s environmental roles are shaped through indigenous cosmology and ritual participation within a non-Western religious context. Third, it contributes to debates concerning religion and sustainability by illustrating how indigenous cosmological systems may sustain everyday environmental discipline through socially embedded moral discipline, collective participation, and sacred ecological ethics.

More broadly, the Kajang case offers important sociological insights into how indigenous religious systems may contribute alternative approaches to environmental sustainability amid the growing limitations of technocratic governance models. At a time when ecological crises increasingly expose the fragility of externally imposed environmental management, the Kajang community demonstrates how sustainability may emerge through culturally grounded moral systems religion, ecology, gender, and collective social life.

## 2 Literature review and theoretical framework

### 2.1 Religion and environmental sustainability

The relationship between religion and environmental sustainability has become an increasingly significant area of inquiry within contemporary social science, particularly in response to intensifying global ecological crises such as climate change, deforestation, biodiversity loss, and environmental degradation (Jenkins, 2009; Jenkins et al., 2018; White, 1967). Earlier scholarship frequently portrayed religion especially monotheistic traditions as contributing to anthropocentric worldviews legitimizing human domination over nature (White, 1967). However, subsequent developments within religion and ecology scholarship have increasingly challenged this assumption by demonstrating that religious systems may also function as important sources of ecological ethics, environmental responsibility, and sustainability-oriented moral practice (Sponsel, 2012; Taylor, 2009; Tucker and Grim, 2001).

Recent scholarship increasingly emphasizes that religion influences environmental behavior not only through formal doctrine or institutional authority but also through culturally embedded systems of meaning shaping everyday ecological conduct (Veldman et al., 2014). Religious values frequently organize collective understandings of environmental obligation, ecological restraint, and moral responsibility toward non-human life. Environmental preservation therefore emerges not merely as a technical or policy-oriented concern but also as a socially embedded moral practice linked to collective identity, ritual participation, and cosmological belief.

At the same time, critical environmental scholars increasingly argue that ecological crises cannot be adequately understood through technocratic governance and state-centered environmental regulation alone (Dryzek, 2021; Escobar, 2018). Sustainability practices are also shaped through locally grounded moral systems, culturally situated ecological knowledge, and socially embedded forms of environmental governance. Consequently, scholars have increasingly emphasized the importance of indigenous ecological systems and sacred ecology in sustaining environmental continuity through ritual discipline, customary norms, and collective participation (Berkes, 2017; Grim and Tucker, 2014).

### 2.2 Lived religion and everyday ecological practice

The concept of lived religion provides an important framework for understanding how religion operates beyond formal institutions, doctrinal systems, and official religious authority. Rather than treating religion primarily as theological belief or institutional structure, lived religion scholarship examines how religious meaning is reproduced through embodied practice, ordinary routines, material interaction, and everyday moral conduct (Ammerman, 2021; McGuire, 2008; Orsi, 2016).

From this perspective, religion is enacted through practical activities shaping how individuals experience morality, social obligation, identity, and collective life within ordinary social contexts. Religious meaning therefore emerges not solely within sacred institutions or ritual events but also through daily practices such as caregiving, work, social interaction, and environmental engagement. This perspective significantly expands sociological understandings of religion by emphasizing embodiment, relationality, and ordinary social experience as central dimensions of religious life (Vásquez, 2011).

Recent developments in lived religion scholarship further emphasize that religion should be understood as socially situated practice continuously reproduced through routine participation and moral negotiation rather than fixed doctrinal systems alone (Ammerman, 2021). Such approaches are particularly important for examining indigenous societies in which distinctions between religion, morality, cosmology, and social organization are often fluid and interconnected.

Applying lived religion to environmental sustainability enables analysis of how ecological responsibility becomes integrated into everyday religious and moral practice. Environmental preservation may therefore function not only as practical resource management but also as embodied religious action reproduced through ritual participation, collective discipline, and socially shared moral expectations.

Despite its analytical significance, lived religion scholarship has only recently begun to engage environmental sustainability as a central area of inquiry. Existing studies frequently focus on domestic spirituality, ritual life, identity formation, or everyday morality while giving less attention to how ecological governance itself may operate through lived religious systems. This study contributes to these discussions by examining how environmental sustainability within the Kajang community is reproduced through everyday religious practice embedded within ritual participation, cosmological belief, and ecological discipline.

### 2.3 Ecofeminism and gendered ecological responsibility

Ecofeminism provides a critical framework for analyzing the relationship between gender, ecology, and systems of power (Shiva and Mies, 2014). Early ecofeminist scholarship emphasized the interconnected domination of women and nature within patriarchal social systems privileging control, hierarchy, and exploitation over relationality and care (Merchant, 1983, 2006; Plumwood, 1993; Shiva, 1988). Within this perspective, women frequently occupy central positions in ecological care due to historically gendered relationships connecting subsistence, caregiving, reproduction, and environmental responsibility.

More recent ecofeminist scholarship has complicated earlier essentialist tendencies by emphasizing intersectionality, cultural specificity, and the importance of socially situated environmental practices (Arora-Jonsson, 2011). Gendered ecological roles are therefore understood not as universal biological characteristics but as socially and culturally produced forms of moral responsibility shaped through religion, history, class, ethnicity, and local cosmological systems.

Contemporary ecofeminist scholars increasingly argue that ecological care must be analyzed within culturally grounded systems of meaning rather than universalized assumptions concerning women’s relationship with nature (Agarwal, 2010; Gaard, 2015). This perspective opens important possibilities for examining how women’s environmental participation is shaped through indigenous cosmologies, ritual systems, and lived religious practice.

Nevertheless, much ecofeminist scholarship continues to be shaped primarily by secular and Western intellectual frameworks. Consequently, relatively limited attention has been given to how ecological responsibility operates within indigenous religious systems and non-Western cosmological contexts. Women’s environmental roles are frequently interpreted primarily as labor, subsistence activity, or caregiving practice rather than as forms of religious authority embedded within collective moral systems.

This study addresses this limitation by examining how women’s ecological participation within the Kajang community operates simultaneously as environmental practice, ritual responsibility, and lived religious authority. In this context, women function not merely as environmental caretakers but as religious-ecological actors involved in sustaining cosmological continuity, moral discipline, and ecological governance within everyday communal life.

### 2.4 Indigenous cosmology and sacred ecology

Indigenous cosmology constitutes an important framework for understanding how environmental relations are embedded within systems of spiritual meaning, ritual obligation, and collective moral order. In many indigenous societies, nature is understood not as an external object or exploitable resource but as part of a relational cosmological system connecting humans, ancestors, spirits, and non-human life within a shared moral universe (Descola, 2013; Ingold, 2021).

Within these cosmological systems, forests, rivers, mountains, and ecological spaces frequently possess sacred significance. Environmental preservation therefore becomes inseparable from ritual discipline, customary law, and collective moral responsibility (Rappaport, 1999; Taylor, 2009). Violations of ecological norms may simultaneously represent social, spiritual, and environmental disruption.

Recent scholarship on sacred ecology further demonstrates that indigenous customary ecological governance often operates through internalized moral systems reinforced by ritual participation, symbolic meaning, and collective social expectation rather than formal bureaucratic regulation alone (Berkes, 2017; Escobar, 2018). Ecological sustainability is therefore reproduced through socially embedded cosmological frameworks organizing everyday environmental behavior and communal ecological responsibility.

These perspectives challenge dominant modern distinctions separating nature, religion, and society by emphasizing relational forms of ecological existence connecting humans and non-human life within morally structured cosmological systems (Kohn, 2013). Indigenous cosmologically embedded governance consequently emerges not merely as resource management but as part of a broader moral and spiritual organization of collective life. This perspective is especially relevant for understanding the Kajang community, where environmental preservation is closely connected to *Pasang ri Kajang* and the cosmological concept of *anrongta*. Within this system, forest protection operates not simply through practical conservation strategies but through ritualized moral discipline, collective participation, and sacred ecological obligation embedded within everyday social life.

### 2.5 Research gap and integrated analytical framework

Although substantial scholarship exists concerning religion and ecology, lived religion, ecofeminism, and indigenous cosmology, these discussions are often developed separately. Existing studies on religion and sustainability frequently emphasize institutional ethics or theological discourse while giving comparatively limited attention to everyday religious practice and indigenous ecological systems. Similarly, ecofeminist scholarship has often insufficiently addressed the role of cosmological belief and lived religion in shaping women’s ecological participation within non-Western indigenous contexts.

As a result, relatively limited scholarship has examined how religion, gender, cosmology, and customary ecological governance operate simultaneously as mutually embedded dimensions of ordinary social life. This limitation is particularly visible in studies of indigenous environmental governance, where ecological sustainability is frequently interpreted primarily through cultural tradition or ecological knowledge while neglecting religion as an active mechanism of moral regulation and social organization.

To address these limitations, this study develops an integrated analytical framework combining lived religion, ecofeminism, and indigenous cosmology. The concept of lived religion provides a framework for analyzing how ecological responsibility is reproduced through ordinary practice, embodied discipline, and routine social interaction (Ammerman, 2021; McGuire, 2008). Ecofeminism contributes analytical attention to gendered ecological participation, relational ethics, and forms of moral authority embedded within everyday care practices (Agarwal, 2010). Indigenous cosmology explains how environmental relations are organized through symbolic systems linking morality, ritual obligation, and sacred ecological order (Berkes, 2017).

Rather than treating religion, gender, and ecology as separate analytical domains, this framework conceptualizes ritual ecological practice as a socially embedded process emerging through the interaction of cosmological belief, ritual participation, gendered moral responsibility, and everyday ecological discipline. This integrated approach enables analysis of how sustainability within the Kajang community is reproduced not primarily through external environmental regulation but through lived religious practice embedded within collective social life.

Through this framework, women’s ecological participation can be understood not merely as environmental labor but as a form of decentralized religious authority reproduced through ritual continuity, intergenerational moral transmission, and socially embedded ecological stewardship. At the same time, environmental sustainability emerges as a form of sacred ecology sustained through cosmological meaning, communal participation, and culturally grounded moral obligation.

### 2.6 Figure description and analytical interpretation

**Figure 1:**
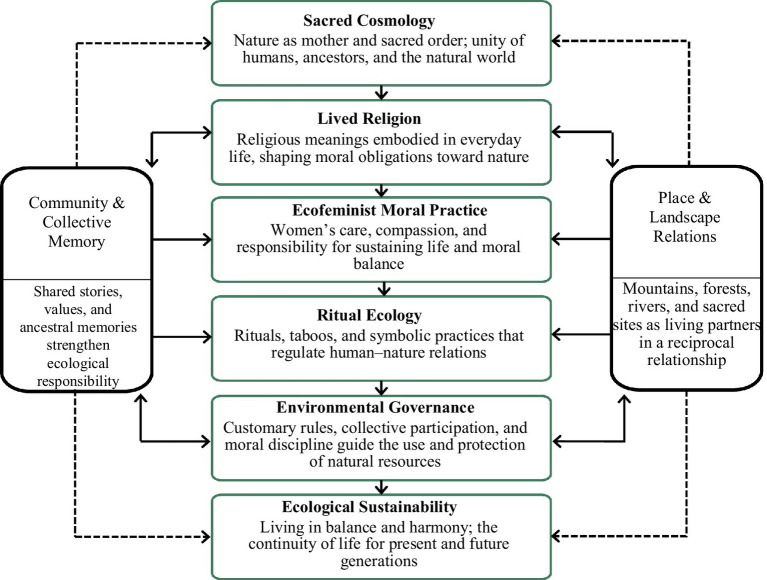
Analytical framework of sacred ecology and lived environmental governance.

Figure 1 presents the integrated analytical framework developed in this study to explain how environmental governance and ecological sustainability are reproduced through the interaction of indigenous cosmology, lived religion, gendered ecological authority, and collectively embedded moral practice within the Kajang community. Rather than depicting environmental governance as a formal institutional arrangement, the framework conceptualizes sustainability as an outcome of socially embedded religious-ecological relations that are continuously reproduced through everyday life.

At the foundation of the framework is indigenous cosmology, represented by the Kajang understanding of anrongta, which positions nature as a sacred maternal entity and situates humans, ancestors, and the natural world within a shared moral order. This cosmological orientation provides the ethical basis through which environmental preservation acquires religious legitimacy and collective social meaning. Nature is therefore not perceived merely as a material resource but as a living relational presence requiring reciprocity, restraint, and moral responsibility.

Building upon this cosmological foundation, lived religion functions as the mechanism through which cosmological meanings become embodied in everyday ecological practice. Religious commitment is expressed not only through formal ritual activity but also through ordinary forms of environmental conduct, social interaction, and moral discipline. Ecological responsibility is therefore enacted through routine practices that integrate religious meaning into daily life and reinforce collective understandings of appropriate relations between humans and nature.

The framework further highlights the importance of ecofeminist moral practice, represented by women’s participation in ecological stewardship, ritual continuity, domestic environmental regulation, and intergenerational transmission of customary values. Women occupy significant positions within the reproduction of religious-ecological life because they sustain the moral and cultural processes through which ecological responsibility is learned, practiced, and transmitted across generations. Their authority emerges primarily through socially recognized moral influence and everyday participation rather than through formal institutional leadership structures.

The interaction between lived religion and ecofeminist moral practice generates ritual ecology, understood as the system of ritual obligations, ecological taboos, symbolic practices, and customary disciplines that regulate human engagement with the environment. Through ritual ecology, cosmological beliefs are translated into practical forms of ecological conduct and collective environmental responsibility. Rituals therefore function not only as symbolic expressions of belief but also as mechanisms of ecological regulation and moral reinforcement.

At the governance level, environmental regulation emerges through customary norms, collective participation, ritual obligations, ecological restraint, and socially internalized moral discipline. Governance within the Kajang community is therefore embedded within everyday social life rather than operating primarily through bureaucratic institutions or externally imposed environmental policies. Environmental authority derives from collective legitimacy, cosmological obligation, and shared moral expectations that shape ecological behavior across the community.

The framework ultimately leads to ecological sustainability, understood as the maintenance of balance, continuity, and harmonious coexistence between humans and the natural world. Sustainability is not conceptualized solely as an environmental outcome but as the cumulative result of ongoing interactions between cosmology, religion, ritual practice, gendered moral participation, and communal ecological responsibility.

Two contextual dimensions further reinforce the entire framework. Community and collective memory sustain ecological responsibility through ancestral narratives, shared values, and intergenerational transmission of moral knowledge. At the same time, place and landscape relations emphasize the significance of forests, rivers, mountains, and sacred ecological spaces as living sites through which religious meaning and environmental responsibility are continuously reproduced. These contextual dimensions demonstrate that environmental governance within the Kajang community is inseparable from the historical, cultural, and spatial relationships through which sacred ecology is maintained.

Taken together, the framework illustrates that environmental sustainability within the Kajang community operates as a form of sacred ecology in which cosmological belief, lived religious practice, women’s ecological authority, ritual discipline, and communal moral participation function as mutually reinforcing dimensions of environmental governance. The model contributes to scholarship on religion and ecology by demonstrating how sustainability may emerge through culturally embedded systems of meaning and everyday religious practice rather than through institutional regulation alone.

## 3 Methods

### 3.1 Research design and approach

This study employs a qualitative interpretive design using a phenomenological approach to examine how religion, gender, and ecological responsibility are experienced and reproduced within the everyday life of the Kajang indigenous community in South Sulawesi, Indonesia. A qualitative approach was selected because the study seeks to understand socially embedded meanings, ritual participation, moral discipline, and lived ecological practices that cannot be adequately captured through quantitative measurement alone (Creswell and Poth, 2016; Patton, 2014).

Phenomenology provides an important analytical framework for examining how individuals interpret and experience reality through embodied participation, culturally situated knowledge, and everyday social interaction (Bantugan, 2025; van Manen, 2007). This approach is particularly relevant for the study of lived religion because it enables exploration of how religious meaning operates through ordinary practice, ritual participation, ecological conduct, and moral obligation embedded within communal life (Ammerman, 2021; McGuire, 2008).

The study also draws upon interpretive sociology, which understands social reality as constructed through shared meaning, symbolic interaction, and collective moral systems (Pervin and Mokhtar, 2022; Schwartz-Shea and Yanow, 2013; Schwartz-Shea, 2014). Within the Kajang community, environmental governance is not merely a formal institutional arrangement but a socially embedded moral system organizing relationships between humans, nature, ancestors, and collective life. Accordingly, this research prioritizes participants’ lived experiences and situated interpretations in understanding how ecological sustainability is reproduced through religiously grounded social practice.

In addition, this study adopts an abductive analytical orientation connecting empirical findings with broader theoretical discussions concerning lived religion, sacred ecology, ecofeminism, and indigenous cosmology (Tavory and Timmermans, 2014). Rather than imposing rigid theoretical categories upon the field data, the analysis moved iteratively between empirical observation and conceptual interpretation in order to generate theoretically grounded sociological explanations.

### 3.2 Research site and context

The research was conducted within the Kajang indigenous community located in Bulukumba Regency, South Sulawesi, Indonesia. The Kajang community represents one of Indonesia’s indigenous societies that continues to maintain strong customary traditions, ritual continuity, and environmentally grounded cosmological systems amid broader processes of modernization and social transformation.

The community is widely recognized for its adherence to *Pasang ri Kajang*, a customary moral system regulating ritual life, social conduct, and ecological relations. The Kajang belief system, known as Patuntung, is fundamentally governed by Pasang ri Kajang, which is understood as a collection of sacred mandates from ancestors and God (Turie’ A’ra’na) (Arifin et al., 2011; Nurfatimah et al., 2025). Patuntung emphasizes the pursuit of truth through three main pillars: reverence for God, the land, and ancestors (Lullulangia et al., 2021; Sahabuddin and Hildayanti, 2023). Pasang functions as a moral and customary legal guide that regulates macro and microcosmic relationships to ensure harmony between humans, nature, and the creator (Arifin et al., 2011; Ridhoh and Alfian, 2025). Core values such as honesty (lambusu), patience (sa’bara), and simplicity serve as the character foundation that every community member must uphold (Erawati et al., 2022). Within this cosmological framework, forests are treated as sacred ecological spaces that must be protected through collective moral discipline, ritual obligation, and customary law. Environmental preservation therefore operates not only as ecological management but also as a form of sacred moral responsibility embedded within everyday communal life.

The Kajang case provides an important context for examining how religion, gender, cosmology, and environmental governance intersect within indigenous societies. The community also offers a significant example of how locally grounded ecological ethics continue to shape environmental sustainability outside dominant technocratic governance frameworks.

### 3.3 Data collection

Fieldwork was conducted over a two-month period (February–March 2024) through in-depth interviews, participant observation, and documentation. This two-month intensive fieldwork period was embedded within a broader ten-month research process (September 2023–June 2024) that encompassed research design, literature review, instrument development, fieldwork execution, data analysis, and manuscript writing. Data collection focused on understanding how ecological responsibility, ritual participation, cosmological belief, women’s ecological authority, and everyday religious practice are experienced and reproduced within the Kajang indigenous community.

Participant selection was conducted using a purposive sampling technique with a snowball sampling approach, starting from a key figure (gatekeeper), namely Ammatoa, as the highest customary leader, and then expanding to other customary stakeholders (Karaeng Tallua, Ada’ Limayya), female customary officials (Anrongta), customary elders, and regular community members. The recruitment process involved coordination with the Head of Tana Toa Village and local customary officials to ensure the hierarchical representation of the Kajang community’s social structure.

The total of 27 participants was determined based on the principle of thematic saturation, whereby sampling was halted when no new information or themes emerged from the interviews (Braun and Clarke, 2021; Guest et al., 2006). Saturation was achieved after the interview with the 23rd participant; however, to enrich the perspectives of the younger generation, 4 youth participants (aged 18–25 years) were added, bringing the total to 27 participants. This addition ensured cross-generational representational coverage in understanding the dynamics of customary-based environmental governance.

The demographic composition of the participants consisted of: 1 Ammatoa; 3 Karaeng Tallua (Karaeng Labbiria, Sulehatang, Moncong Buloa); 10 customary stakeholders from Ada’ Limayya (Galla Pantama, Galla Lombo, Galla Malleleng, Galla Puto, Galla Kajang, Galla Anjuru, Galla Sangkala, Galla Sapa, Galla Bantalang, Galla Ganta); 4 other customary officials (Anrongta ri Pangi, Anrongta ri Bongkina, Sanro Pakrasangang, Anronta); 5 general community members; and 4 additional youth participants. The gender composition included 16 males (59.3%) and 11 females (40.7%), with an age range of 20–72 years.

Semi-structured interviews were conducted face-to-face at each participant’s residence or at locations of rituals/daily activities, with an average duration of 60–120 min per session. Several key participants, such as the Ammatoa and senior customary stakeholders, were interviewed in 2–3 separate sessions to explore the depth of information.

The language used in the interviews was the Konjo dialect of Makassarese, which is the everyday language of the Kajang community. The researcher was accompanied by a local research assistant who is a native speaker of the Konjo language and understands the customary values of Kajang, to ensure accuracy of meaning and to avoid misinterpretation due to dialectal differences. All interview sessions were recorded with a digital recorder after obtaining written consent from each participant.

Interview recordings were transcribed verbatim in the Konjo/Makassar language by the local research assistant, and subsequently translated into Indonesian by the researcher together with the research assistant through a back-translation process to ensure the accuracy of meaning for cultural concepts such as anrongta, Pasang ri Kajang, kamase-mase, and other customary terms. The translation process involved discussions with customary stakeholders to verify the appropriate equivalent terms in Indonesian.

To ensure the validity of the interpretation, member checking was conducted by returning the summary of interview results and preliminary findings to 9 key participants (Ammatoa, 3 Karaeng Tallua, 3 Galla, and 2 customary women) to confirm their alignment with the participants’ understanding. Revisions were made based on participant feedback, particularly regarding the interpretation of customary terms and descriptions of ritual practices. Additionally, peer debriefing discussions were conducted with two other researchers who have research experience in indigenous communities of South Sulawesi, to examine the robustness of the interpretations and identify potential biases in the analysis.

The interviews explored participants’ understandings of environmental preservation, ritual obligations, cosmological beliefs, women’s ecological roles, customary norms, and lived experiences of environmental stewardship. Particular attention was given to how participants interpreted the relationship between nature, ancestral teachings, religious obligations, and everyday ecological conduct. The semi-structured format allowed participants to elaborate on their experiences while enabling the researcher to explore emerging themes relevant to the study’s analytical focus.

Participant observation was conducted throughout the fieldwork period during ritual activities, communal gatherings, agricultural practices, and routine environmental interactions. Observation enabled a deeper understanding of how ecological discipline, moral obligations, and religious meanings were enacted through everyday participation and social practice. Prolonged engagement within the community further allowed the researcher to observe how environmental responsibility was reproduced through collective interaction, customary discipline, and routine social participation rather than through formal institutional regulation alone (Emerson et al., 1995).

Documentation included customary narratives, ritual regulations, community records, local oral traditions, and secondary literature concerning Kajang cosmology, indigenous environmental governance, religion, and sustainability practices. These materials were used to strengthen contextual interpretation and support analytical triangulation across multiple sources of evidence.

Data collection proceeded iteratively throughout the fieldwork period. Emerging insights from interviews informed subsequent observations and documentary analysis, while observations and documentary materials were continuously compared with participant narratives to deepen contextual understanding. Fieldwork continued until thematic saturation was reached and no substantially new themes emerged concerning lived religion, ecological responsibility, ritual participation, women’s moral authority, and sacred ecological governance (Guest et al., 2020; Saunders et al., 2018).

### 3.4 Data analysis

Data were analyzed using thematic analysis following Braun and Clarke. Although this study employs thematic analysis as a technical procedure, the entire analytical process is informed by phenomenological principles to ensure that the resulting themes reflect the participants’ lived experiences, rather than being merely constructed from the researcher’s theoretical categories. The relationship between phenomenology and thematic analysis in this study is operationalized through the following strategies.

First, the coding process was conducted inductively, with initial codes emerging directly from the data (interview transcripts, field notes, and documents) rather than being derived from a theoretical framework (Braun and Clarke, 2006). The researcher allowed the data to “speak” by reading transcripts repeatedly and noting phrases, expressions, and concepts articulated by participants in their own words. This inductive approach aligns with the phenomenological principle of understanding phenomena from the perspective of the subjects who experience them.

Second, the researcher applied the principle of bracketing throughout the coding and theme development process. Prior to beginning the analysis, the researcher wrote reflective memos documenting initial assumptions, the theoretical frameworks used as sensitizing concepts (rather than as fixed categories), and expectations regarding the findings. These memos were periodically reviewed during the analysis to identify and suspend the influence of potential biases arising from the researcher’s prior knowledge or experience.

Third, the process of eidetic reduction was conducted by comparing and contrasting the experiences of various participants to identify the fundamental structures of the phenomenon under study (van Manen, 2007). For example, the theme of anrongta as a sacred cosmology did not emerge from prior theoretical definitions, but rather from the repetition of participants’ expressions across various contexts from customary leaders, women, elders, to youth who consistently described the forest as a “mother” that both protects and requires protection.

Fourth, the researcher employed imaginative variation by consciously considering alternative perspectives and counter-possibilities to each emerging theme. This strategy prevented the analysis from becoming too narrow or merely confirming dominant narratives, while also opening space for the complexity and ambiguity of participants’ experiences.

To ensure transparency of the analytical process, the entire analytical trail from initial codes, code grouping, to theme development was systematically documented and made available for the audit process (Lincoln and Guba, 1985). This enables readers and reviewers to trace how the final themes genuinely emerged from the data, rather than from the researcher’s theoretical framework.

Operationally, the thematic analysis was conducted through several stages. The first stage was data familiarization, during which all interview transcripts, field notes, and documents were read repeatedly to build a deep understanding of the participants’ experiences. At this stage, initial analytical notes were made to record recurring ideas, symbolic expressions, ritual practices, and ecological meanings emerging from the data.

The second stage was initial coding. Codes were generated inductively with a focus on references to cosmological beliefs, ritual obligations, ecological management, gender participation, customary norms, environmental regulation, and the relationship between religious practices and ecological behavior. Particular attention was paid to how participants described the moral meaning of the forest, ancestral teachings, environmental responsibility, and everyday interactions with nature.

The third stage was grouping related codes into broader thematic categories. Through continuous comparison across data sources, several main themes emerged, including sacred cosmology and the environmental moral order, ritual ecological discipline, women’s religious-ecological authority, simplicity and ecological restraint, and lived religion as embedded environmental governance. These themes were further refined through repeated engagement with the empirical materials to ensure that they accurately reflected the participants’ experiences and social realities.

The analysis further employed an abductive orientation that moved iteratively between empirical observation and theoretical interpretation (Tavory and Timmermans, 2014). Rather than imposing predetermined theoretical categories upon participants’ experiences, theoretical concepts derived from lived religion, ecofeminism, sacred ecology, and indigenous cosmology were used as sensitizing frameworks through which emerging patterns could be interpreted and contextualized. This recursive process enabled the researcher to connect local experiences and practices within the Kajang community to broader sociological discussions concerning religion, ecology, gender, and environmental governance.

Analytical attention was directed not only toward observable ecological behavior but also toward the symbolic meanings, moral assumptions, and cosmological understandings through which environmental responsibility was socially organized and reproduced. This approach was particularly important because environmental governance within the Kajang community operates through culturally embedded systems of meaning rather than through formal institutional structures alone.

To strengthen analytical credibility, triangulation was conducted across interviews, participant observation, and documentary materials (Denzin, 2017). Themes identified in one source were continuously compared with evidence from other sources to ensure interpretive consistency and analytical depth. This process enabled the study to develop a comprehensive understanding of how sacred ecology, lived religion, indigenous cosmology, and gendered moral participation interact in shaping environmental governance within everyday Kajang social life.

### 3.5 Reflexivity and positionality

Because this study involves indigenous cosmology, customary knowledge, and lived religious practice, reflexivity constituted an important dimension of the research process. The researcher remained aware that interpretation is shaped through social position, cultural interaction, and field engagement rather than detached neutrality alone (Baily, 2025; Berger, 2015).

Particular attention was therefore given to maintaining sensitivity toward local cultural norms, ritual boundaries, and community-based systems of meaning during both fieldwork and interpretation. Rather than reducing Kajang ecological practices into external analytical categories alone, the study sought to understand environmental responsibility from the perspective of participants’ lived moral and cosmological experiences.

The research process also involved continuous reflection concerning researcher positionality, interpretive authority, and cross-cultural representation, particularly in relation to indigenous knowledge systems and environmentally embedded religious practice.

### 3.6 Research ethics

This study received ethical approval from the Academic Board of the Faculty of Social Sciences and Law, Universitas Negeri Makassar (Approval No: 192/UN36.6/KP/2024, dated February 27, 2024). All participants were adults aged 18 years and above; no minors were involved. Written informed consent was obtained from each participant, with verbal consent and community witness for non-literate participants, following culturally appropriate procedures (Smith, 2012).

Throughout the research process, the researcher maintained ongoing consultation with customary leaders to respect communal values, ritual restrictions, and culturally sensitive knowledge. Anonymized quotations (e.g., AM-001, WT-005) protect participant confidentiality. Raw data are securely stored and accessible only to the research team. Preliminary findings were shared with community representatives prior to publication to ensure cultural appropriateness.

## 4 Findings

### 4.1 Sacred cosmology and environmental moral order

The findings demonstrate that environmental preservation within the Kajang community is deeply embedded in cosmological beliefs that define the relationship between humans and nature as moral, spiritual, and relational rather than merely material. Participants consistently described the forest not simply as a source of livelihood or economic resource but as a sacred ecological space connected to ancestral teachings, ritual obligations, and collective moral order. Within this cosmological system, environmental governance operates through sacred ecological ethics embedded in everyday communal life.

Central to this worldview is the concept of *anrongta*, which positions nature as a nurturing mother requiring protection, restraint, and reciprocal moral responsibility. From an ecotheological perspective, the Kajang people view nature through the concept of anrongta, where the earth and forests are considered as “mother” that provides life and must therefore be spiritually protected (Ardilla et al., 2025; Ridhoh and Alfian, 2025). This nature conservation practice is manifested through a strict forest zoning system: Borong Karama (sacred forest) which is forbidden for all activities except rituals, Borong Batasayya (border forest) with limited utilization upon Ammatoa’s permission, and Borong Luarayya (people’s forest) (Lullulangia et al., 2021; Ridhoh and Alfian, 2025). The principle of kamase-masea or simplicity serves as a consumption control mechanism to maintain ecosystem balance (Haq et al., 2024; Nurfatimah et al., 2025). Violations of this zoning system are subject to customary sanctions known as ba’bala, which include physical caning to social ostracism (Ridhoh and Alfian, 2025; Sartini et al., 2026). Through this cosmological understanding, environmental preservation becomes a sacred obligation rather than solely an ecological or economic concern. Participants frequently explained environmental care through relational language emphasizing kinship, reciprocity, and moral responsibility toward both ancestors and future generations.

One community elder explained:

“The forest is not only ours. It belongs to our ancestors and to future generations. If we destroy it, we also destroy our moral balance.”

This statement illustrates how ecological responsibility is understood as part of a broader moral relationship connecting humans, ancestors, and ecological continuity. Environmental damage is therefore interpreted not merely as material destruction but as a disruption of social, spiritual, and cosmological harmony. The forest occupies a position within a larger moral universe in which ecological care reflects respect for ancestral teachings and collective obligations extending across generations.

Another participant similarly stated:

“Nature protects us because we protect it. If humans become greedy, the balance between people and the forest will disappear.”

This perspective further demonstrates that environmental restraint within the Kajang community is grounded in principles of reciprocity rather than resource management alone. Participants repeatedly emphasized that ecological well-being depends upon maintaining balance between human needs and the moral obligations associated with protecting the natural world. Environmental preservation therefore functions simultaneously as ecological practice, social responsibility, and religious duty.

Field observations further revealed that cosmological meanings are continuously reproduced through oral transmission, ritual participation, customary teachings, and everyday ecological conduct. Knowledge concerning appropriate relations with nature is communicated not only through formal ritual settings but also through routine social interaction, family instruction, and participation in communal activities. Religion is therefore not separated from environmental practice but operates through embodied forms of social participation that shape ecological behavior within everyday life.

The findings additionally indicate that sacred ecology functions as a socially effective governance system because environmental ethics are embedded within collective moral consciousness rather than dependent solely upon external regulation. Ecological sustainability is maintained through internalized moral obligations reinforced by ancestral authority, communal expectations, and cosmological understandings of environmental balance. In this sense, environmental governance within the Kajang community emerges through a lived moral order in which ecological responsibility is inseparable from cosmological belief, religious meaning, and collective social life.

The classification of forests in Kajang cosmology reveals a structured system of ecological knowledge. The Kajang community divides forests into three zones, each with distinct functions and regulations. First, Borong Karamaka (sacred forest) an area strictly prohibited for all types of activities, except for ritual ceremonies. This forest is regarded as the dwelling place of the ancestors and the site where Tu Rie A’ra’na (God Almighty) descends. This zone may only be entered by the Ammatoa and customary stakeholders during specific ritual ceremonies. Even planting trees is forbidden in this area.

Second, Borong Batasayya (border/limited production forest) an area that may be utilized under strict conditions: (1) permission must be obtained from the Ammatoa; (2) the number of trees to be felled is determined by the Ammatoa; (3) before cutting down one tree, two trees of the same species must be planted and nurtured until they grow well; and (4) replanting does not necessarily have to be carried out at the felling location. The use of timber from this forest is permitted only for the construction of public facilities and houses for community members who are truly impoverished.

Third, Borong Luarayya (community forest) an area that may be utilized to meet daily livelihood needs. This three-zone system demonstrates that Kajang’s ecological governance is not uniform, but rather varies according to the degree of sacredness and ecological function of each area. Any violation of these boundaries carries firm customary consequences.

Although this study primarily focuses on socio-cultural aspects, there is secondary data that supports the effectiveness of the Kajang customary governance system in maintaining ecological sustainability. The Ammatoa Kajang Customary Forest, covering 313.99 hectares, was designated through Minister of Environment and Forestry Decree Number SK.6746/MENLHK-PSKL/KUM.1/12/2016 as the first customary forest in Indonesia to be officially recognized by the government (ANTARA News, 2017; Ministry of Environment and Forestry, 2016). This designation constitutes state recognition of the rights of indigenous peoples to sustainably manage forests based on local wisdom (Nurkhalis et al., 2020).

A study by Forests, Trees and Agroforestry, involving CIFOR, shows that forests managed by the Kajang indigenous community still contain old-growth forests and high-value economic species, in contrast to state-protected forests in neighboring sub-districts, which have experienced rapid conversion into settlements and non-conservation uses (Forests, 2018). This comparison indicates that the Kajang customary governance system may be more effective in maintaining forest cover compared to the state protection system in the same region.

Ecologically, the Kajang customary forest functions as a conservation area for agriculture and a source of clean water for communities in three watersheds Raowa, Baonto, and Apparang and possesses biodiversity potential in the form of endemic flora and fauna of South Sulawesi (Adaptation Fund, 2024; Nurkhalis et al., 2020). The Kajang community has also received an award from the Minister of Environment and Forestry for being considered capable of caring for the forest through local wisdom (PPID KLHK, 2016). Nevertheless, it must be acknowledged that this study did not collect independent ecological data such as longitudinal forest cover measurements or biodiversity indices.

### 4.2 Ritual practice and ecological discipline

Field observations further demonstrate that ritual practices play a central role in regulating environmental behavior and reinforcing ecological discipline within the Kajang community. Rituals function not merely as ceremonial events or symbolic expressions of tradition but as socially embedded mechanisms through which environmental responsibility is collectively reproduced, remembered, and morally legitimized.

Participants consistently described ritual participation as necessary for maintaining harmony between humans, nature, and ancestral order. Ritual activities frequently accompany agricultural cycles, forest-related practices, and communal decision-making processes. Through repeated participation, ritual practice reinforces collective expectations concerning simplicity, restraint, reciprocity, and ecological responsibility. Rituals therefore operate as practical sites where cosmological beliefs are translated into everyday environmental conduct.

One customary leader explained:

“The rituals remind us that nature cannot be treated carelessly. The forest protects our lives, so we must also protect it.”

This statement illustrates how ritual participation continuously connects environmental conduct with religious obligation and collective moral identity. Ecological responsibility is not understood as an external requirement imposed by regulation but as a sacred commitment reinforced through ritual participation and communal memory. Rituals repeatedly remind community members of their obligations toward both nature and ancestral teachings, thereby strengthening ecological discipline across generations.

Another participant noted:

“When rituals are performed, people remember the teachings of the ancestors. The rituals teach us to live carefully with nature.”

This perspective highlights the role of ritual as a mechanism of moral transmission. Ritual participation does not merely reproduce symbolic traditions but also cultivates ecological consciousness by embedding environmental responsibility within shared systems of sacred meaning. Through ritual repetition, participants learn appropriate forms of conduct, restraint, and respect in their interactions with the natural world.

Field observations further revealed that ritual activities strengthen social cohesion and reinforce collective responsibility for environmental stewardship. Participation in ritual events brings together different generations and social groups, creating opportunities for the transmission of ecological values and customary teachings. Ecological governance within the Kajang community therefore operates not through individualized environmental awareness alone but through communal participation and collectively reinforced moral discipline.

These findings support scholarship on lived religion emphasizing that religion frequently operates through embodied practice, routine participation, and socially situated forms of meaning-making rather than abstract doctrinal instruction alone (Orsi, 2016). In the Kajang context, ritual practice functions as a key mechanism through which sacred ecology is reproduced within everyday social life. Rituals institutionalize ecological responsibility by linking environmental behavior to collective identity, ancestral authority, and shared moral obligations.

At the same time, several participants acknowledged that modernization and external social influences increasingly affect younger generations’ engagement with customary ritual practices. One elder remarked:

“Young people today are more connected to the outside world. Some still follow the traditions strongly, but others are beginning to forget the ancestral teachings.”

This observation suggests that sacred ecological governance within the Kajang community continues to be negotiated amid broader processes of social transformation. While ritual practices remain socially influential, their long-term continuity depends upon ongoing intergenerational transmission and the ability of customary institutions to maintain their moral relevance within changing social conditions.

Customary rituals in the Kajang community are not merely ceremonial but have structured and binding procedures. One of the most central rituals is Andingingi, which means “cooling” a ritual to pray to the Almighty for continued safety in managing natural resources (Nurfadillah et al., 2023) such as agriculture, plantations, and others. This ritual is believed to be capable of bringing rain to irrigate agricultural land, thereby optimizing harvests.

Within the agricultural cycle, there is the Pa′tumbu ritual, which is performed twice: before planting begins and 1 week before the harvest. This ritual serves as a collective reminder that the relationship between humans and the land is a sacred one that requires respect through customary procedures.

Rituals related to the life cycle also have structured stages. The Akkattere tradition (hair-cutting ritual), for example, begins with apparungrungi (dressing in customary attire), Appacidong Ada’ (seating the customary order), Akkatto Salahi (cutting the necklace), Akkattere (shaving/cutting the hair), Abbaca Doang (reciting prayers), Dedde’ (forming the songkolo), and concludes with the distribution of dallekang (offerings/ceremonial food portions). Each stage holds symbolic meaning that connects the individual with ancestors, the community, and nature.

The annual ritual of Akbattasa Jera (cleaning the graves) is performed on the 22nd day of Ramadan, during which the Ammatoa, along with several trusted individuals, visits the graves at Jera Tunggala, located within the karrasayya forest (sacred forest). Only those with high spiritual knowledge are permitted to participate in rituals within this customary forest, indicating the existence of a hierarchy of sacred knowledge that governs access to certain ecological spaces. This ritual is subsequently followed by baca doang (reciting prayers) at each individual’s home.

Ecological governance within the Kajang community is reinforced by a structured system of customary sanctions. Violations of the Pasang ri Kajang are subject to sanctions known as ba’bala. This sanction system is divided into three levels: (1) Ca′pa Ba′bala (minor sanction), consisting of whipping and a fine of approximately IDR 400,000; (2) Tangnga Ba′bala (moderate sanction); and (3) Pokok Ba′bala (serious sanction), with fines reaching IDR 1,200,000 to IDR 12,000,000 per tree.

There are four main prohibitions in the customary law of Ammatoa Kajang, namely: (1) Tabbang Kaju (cutting trees), (2) Rao doing (taking shrimp and river fish within the area), (3) Rao bani (taking bees within the customary area), and (4) taking rattan within the forest area. If these rules are violated, the perpetrator will be subject to a sanction called “pasang” by the inner Kajang community.

The process for determining sanctions is conducted through a customary deliberation mechanism called A’borong, in which all Galla and customary stakeholders are convened to deliberate. The decisions resulting from this deliberation are then communicated to the entire community. Customary sanctions are not physically coercive. If someone refuses or is unwilling to accept the sanction, they will not be forced; however, the consequence is that the person is no longer considered part of the customary community. The distribution of fine money to all those present in the customary procession is intended to instill collective responsibility among every community member to jointly protect the forest.

Nevertheless, the enforcement of customary rules faces challenges. Several cases indicate a weakening of compliance with customary rules, including cases of encroachment into customary forests, logging by village elites, and land parcelling within the customary forest area. The younger generation has begun to lose belief in ancestral sanctions or supernatural powers, so that if someone refuses a customary hearing, there is no coercive force from the customary system.

### 4.3 Women as religious-ecological authorities

One of the most significant findings of this study concerns the central role of women in sustaining ecological continuity, ritual life, and moral discipline within the Kajang community. Women function not only as participants in environmental activities but also as important religious-ecological actors who contribute to the transmission of cosmological knowledge, the reproduction of ritual continuity, the regulation of everyday ecological conduct, and the maintenance of communal moral values.

Women participants consistently emphasized that environmental responsibility is inseparable from moral education, ancestral teachings, and everyday religious practice. Ecological stewardship is reproduced primarily through routine social interaction, family-based moral guidance, and participation in customary life rather than through formal institutional authority structures. In this context, women play a crucial role in ensuring that ecological values remain embedded within everyday social relations and intergenerational learning processes.

One female participant explained:

“Since childhood we are taught to respect the forest because it protects our lives. Women must teach children how to live properly with nature.”

This statement illustrates how women contribute to the transmission of ecological ethics by connecting environmental responsibility to moral education and communal identity. Ecological knowledge is not acquired solely through formal instruction but is reproduced through everyday interactions within households and family networks where women frequently occupy central educational roles.

Another woman similarly stated:

“Protecting the forest is part of protecting the teachings of our ancestors. Mothers teach these values inside the household.”

This perspective demonstrates that women’s ecological participation extends beyond environmental care itself. Women act as mediators of cosmological continuity by transmitting ancestral teachings, reinforcing moral obligations, and sustaining collective understandings of appropriate relationships between humans and nature. Their contribution is therefore simultaneously ecological, cultural, and religious.

Field observations further revealed that women actively participate in ritual preparation, domestic ecological regulation, and the maintenance of customary practices that support environmental stewardship. Through these activities, women contribute to the continuity of sacred ecological values within everyday communal life. Their influence is exercised through socially recognized responsibilities and practical participation rather than through formal positions of institutional leadership.

Importantly, women’s authority within the Kajang community should not be understood through conventional institutional models of religious leadership. Gender roles in Kajang governance reveal a unique authority structure through the position of Anrongta (Nurjayanti, 2023). Although the highest customary leadership (Ammatoa) is held by men, Anrongta holds central authority as the primary advisor, installer of Ammatoa, and holder of a crucial role in customary deliberations or A’borong (Nurjayanti, 2023; Ridhoh and Alfian, 2025). Women bear full responsibility for the transmission of moral values across generations and the regulation of household economics based on the principle of simplicity (Nurjayanti, 2023; Ridhoh and Alfian, 2025). This synergy between masculine authority in law enforcement and feminine authority in value nurturing creates a robust cultural defense system in maintaining environmental sustainability (Nurfadillah et al., 2023; Nurjayanti, 2023). Instead, it operates through decentralized forms of moral influence embedded within routine participation, ritual continuity, caregiving practices, and intergenerational transmission of ecological knowledge. Their authority emerges from their role in sustaining the moral and cosmological foundations upon which environmental responsibility depends.

These findings contribute to ecofeminist discussions by demonstrating that women’s ecological roles within the Kajang community are not based upon essentialized assumptions concerning an inherent connection between women and nature. Rather, women’s participation is socially and culturally situated within indigenous cosmology, customary obligations, and lived religious practice. Ecological stewardship therefore emerges through historically embedded moral responsibilities and communal expectations rather than biological determinism.

The findings further suggest that women’s ecological authority is socially recognized because environmental responsibility itself is understood as part of a broader moral and sacred order. Women’s everyday participation in teaching ecological values, organizing ritual activities, and maintaining customary discipline functions as a form of religious-ecological authority that contributes directly to the reproduction of sacred ecology within the community.

At the same time, several participants noted that women’s roles continue to evolve alongside broader processes of social transformation. Younger women increasingly negotiate customary responsibilities alongside formal education, economic participation, and wider social mobility. This indicates that women’s religious-ecological authority is not static but continuously reproduced through ongoing interactions between customary continuity and contemporary social change.

The role of women in the Kajang customary structure is not marginal; rather, it is institutionalized through the position of Anrongta. Within the customary leadership structure, there are two types of Anrongta. The Anrongta baku Atowayya has the following functions: (1) inaugurating the Ammatoa, (2) carrying out special duties assigned by the Ammatoa, and (3) serving as an advisor to the Ammatoa. The Anrongta baku Alloyya is tasked with assisting the Anrongta baku Atowayya in carrying out its duties, facilitating the process of electing the Ammatoa, and preparing the necessary requirements for customary rituals.

The position of the Anrongta is highly significant because when the Ammatoa allinrung (passes away), their duties and office are immediately taken over by the Anrongta until the next Ammatoa is elected. In the A’borong process (customary deliberation), the Anrongta plays an important role in decision-making. The Kajang community also permits women to hold government positions outside the customary structure, provided that the women are considered capable and possess the capacity for the position.

Women’s participation in the preparation of rituals such as Andingingi and Maddangang is concrete evidence of how women have a significant role in maintaining the continuity of customary rituals. Through the role of the Anrongta, women in the Kajang tribe play an important role in preserving traditions and customary rituals. Thus, women’s authority in the Kajang community is not merely informal authority, but rather authority that is institutionalized and structurally recognized.

### 4.4 Everyday simplicity and ecological restraint

Another important finding concerns the role of simplicity, moderation, and restraint in shaping environmental behavior within the Kajang community. Participants consistently described ecological sustainability as inseparable from moral self-discipline regulating everyday patterns of consumption, resource use, and human interaction with the natural environment. Environmental responsibility is therefore practiced not only through ritual participation or customary regulation but also through ordinary forms of conduct embedded within daily life.

Community members emphasized that customary teachings discourage excessive exploitation of nature and encourage moderation as a collective ethical orientation guiding relationships between humans and the environment. The physical manifestation of Kajang cosmology is evident in their traditional architecture. Houses are uniform in design and face West as a spiritual qibla toward Mount Bawakaraeng (Erawati et al., 2022; Sahabuddin and Hildayanti, 2023). Vertically, the house is divided into three parts: Para (attic) for storing heirlooms and food supplies, Kale Balla (house body) for social interaction, and Siring (under the house) for livestock and weaving activities (Hildayanti and Machrizzandi, 2022; Sahabuddin and Hildayanti, 2023). Construction is prohibited from using modern materials such as nails or bricks, as brick firing is considered to excessively damage forests (Arifin et al., 2011; Triadi et al., 2025). House pillars are planted directly into the ground without concrete foundations to symbolize that humans must not be separated from the earth as their origin (Arifin et al., 2011; Lullulangia et al., 2021). Ecological sustainability is understood not primarily as a technical objective but as a consequence of living according to moral principles that value balance, reciprocity, and restraint.

One participant explained:

“We only take what we truly need. If people become greedy, nature will no longer protect us.”

This statement reflects a moral understanding of environmental responsibility in which ecological well-being depends upon limiting human desire and avoiding excessive extraction of natural resources. Participants frequently associated environmental degradation with greed, excess, and the disruption of reciprocal relationships between humans and nature. Ecological restraint therefore functions as a form of moral discipline that regulates everyday environmental conduct.

Another participant similarly remarked:

“Living simply is part of respecting nature. Too much desire destroys balance.”

This perspective further illustrates how simplicity is interpreted not merely as an economic necessity but as an ethical and spiritual orientation toward the natural world. Participants repeatedly emphasized that moderation enables individuals to maintain harmonious relationships with nature, while excessive consumption threatens both ecological continuity and communal moral order.

Field observations revealed that values of simplicity and restraint are reinforced through everyday social interaction, communal expectations, and customary teachings. These values influence decisions concerning resource use, consumption practices, and environmental behavior, thereby ecological responsibility into routine patterns of social life. Sustainability is therefore reproduced through ordinary practices that encourage moderation and discourage environmentally destructive forms of excess.

Importantly, ecological restraint within the Kajang community is closely connected to collective identity and moral belonging. Environmental behavior is not treated as a purely individual matter but as a reflection of one’s commitment to customary values and communal responsibility. Individuals who violate environmental norms may encounter social disapproval because ecological conduct is understood as part of a broader moral relationship linking humans, nature, and collective life.

These findings suggest that environmental sustainability within the Kajang community is sustained through internalized moral systems that shape everyday behavior rather than through formal environmental regulation alone. Simplicity and restraint operate as embodied forms of sacred ecological ethics through which environmental responsibility becomes integrated into ordinary social practice. In this sense, sustainability emerges not only from ecological knowledge or customary rules but also from the cultivation of moral dispositions that regulate human desires and patterns of environmental interaction.

At the same time, several participants acknowledged that external economic pressures, expanding market relations, and changing consumption patterns increasingly challenge traditional values of moderation and restraint. Although customary norms remain influential, ecological ethics continue to be negotiated within broader processes of modernization and social transformation. This suggests that the reproduction of sacred ecological values requires continuous moral and cultural reinforcement in order to remain effective under changing social conditions.

The simplicity of life (kamase-mase) is not merely a lifestyle, but rather a moral principle manifested in various aspects of daily life. The Kajang people reject the use of modern tools and live by the principle of simplicity. In their daily lives, they do not use chairs or other luxury items. The household utensils they use are very simple, mostly made from natural materials.

This principle of simplicity is also reflected in the obligation to go barefoot, as a symbol of being united with the earth where humans begin and end. Black is the color of greatness, which must be worn within the customary area, while red is interpreted as a forbidden color. Every symbol in daily life from the direction of the house facing west, to the house pillars that are planted carries a philosophical meaning that reminds of the relationship between humans, ancestors, and nature.

### 4.5 Lived religion and environmentally embedded governance

Taken together, the findings demonstrate that environmental governance within the Kajang community operates through lived religion embedded in sacred cosmology, ritual participation, gendered moral responsibility, and everyday ecological discipline. Religion is not confined to formal doctrine, institutional authority, or ritual events alone. Rather, it is enacted through ordinary social practices that shape ecological behavior, moral responsibility, and collective understandings of appropriate relations between humans and nature.

The concept of lived religion provides a particularly useful framework for understanding how environmental sustainability is reproduced through routine participation, embodied moral conduct, and socially shared ecological obligations. Across interviews and field observations, participants consistently described environmental responsibility as an integral aspect of communal life rather than as a separate environmental concern. Ecological preservation is therefore experienced not as an external regulatory requirement but as part of everyday religious and moral practice.

The findings further demonstrate that sacred cosmology provides the moral foundation upon which ecological responsibility is organized and legitimized. Through the concept of *anrongta*, environmental preservation acquires religious significance and becomes integrated into broader understandings of reciprocity, restraint, and collective responsibility. Cosmological beliefs are continuously reproduced through ritual participation, customary teachings, and everyday social interaction, thereby linking environmental stewardship to shared systems of meaning and moral obligation.

At the same time, ritual practices function as mechanisms through which cosmological values are translated into practical forms of environmental conduct. Ritual participation reinforces ecological discipline, strengthens collective memory, and reproduces communal commitments to environmental protection. Through ritual repetition, environmental ethics become embedded within everyday life and acquire social legitimacy across generations.

The findings also reveal that women occupy central positions within this religious-ecological system through ritual continuity, ecological stewardship, domestic moral education, and intergenerational transmission of cosmological values. Their participation demonstrates that religious authority may operate through decentralized and socially embedded forms of ecological practice rather than through formal institutional leadership structures alone. Women therefore contribute significantly to the reproduction of sacred ecological values and environmental responsibility within everyday communal life.

Equally important, ecological sustainability is reinforced through practices of simplicity, moderation, and restraint that regulate ordinary patterns of environmental interaction. These moral orientations function as embodied expressions of sacred ecological ethics, shaping how community members engage with natural resources and evaluate appropriate forms of environmental conduct. Sustainability is therefore reproduced not only through ritual and cosmology but also through everyday practices that cultivate ecological responsibility as a moral disposition.

Taken together, these findings suggest that environmental governance within the Kajang community is best understood as a culturally embedded system of sacred ecology in which religion, morality, cosmology, gendered participation, and environmental responsibility are mutually constitutive dimensions of social life. Governance does not operate primarily through bureaucratic regulation or external environmental intervention but through socially internalized systems of meaning that shape ecological behavior and collective responsibility.

More broadly, the Kajang case challenges analytical approaches that separate religion, ecology, gender, and governance into distinct domains of inquiry. Instead, the findings demonstrate how environmental sustainability may emerge through relational systems that integrate cosmological belief, ritual practice, women’s moral participation, and everyday ecological discipline within ordinary communal life. This perspective highlights the importance of understanding sustainability not merely as an environmental outcome but as an ongoing socio-religious process reproduced through lived experience, collective participation, and sacred ecological ethics.

Table 1 summarizes the major themes emerging from the empirical findings and demonstrates how environmental governance within the Kajang community is reproduced through the interaction of cosmological belief, ritual participation, gendered moral responsibility, and everyday ecological practice. Rather than representing isolated dimensions of social life, the themes illustrate a mutually reinforcing religious-ecological system through which environmental sustainability is maintained within ordinary communal existence.

**Table 1:** Major themes of religiously embedded environmental governance in the Kajang community.

Major theme	Empirical indicators	Forms of social practice	Sociological interpretation
Sacred Cosmology	Forest understood as sacred ancestral entity	Ecological restraint and forest protection	Cosmology functions as sacred ecological governance
Ritual Ecological Discipline	Ritual participation reinforces environmental obligation	Collective ritual practice and ecological discipline	Ritual practice institutionalizes environmental morality
Women’s Religious-Ecological Authority	Women transmit ecological values across generations	Domestic moral education and ritual continuity	Religious authority operates through gendered everyday practice
Simplicity and Moral Restraint	Limitation of consumption and resource use	Moderation in everyday environmental conduct	Sustainability embedded in moral self-discipline
Lived Religion and Ecology	Ecological preservation practiced as religious obligation	Daily environmental care integrated into ordinary life	Religion functions through embodied ecological practice

The first theme, sacred cosmology, reveals that environmental responsibility is grounded in cosmological understandings that position nature as part of a sacred moral order. Ecological preservation is therefore legitimized through ancestral teachings, collective obligations, and religious meanings that connect environmental conduct to broader systems of moral responsibility. Sacred cosmology functions as the normative foundation upon which environmental governance is constructed.

The second theme, ritual ecological discipline, demonstrates how ritual participation translates cosmological values into practical forms of ecological conduct. Rituals reinforce environmental obligations, strengthen collective memory, and reproduce moral expectations concerning appropriate relationships between humans and nature. Ecological discipline is therefore sustained not only through belief but also through embodied participation and ritual continuity.

The third theme highlights women’s religious-ecological authority. The findings show that women contribute significantly to the transmission of ecological values, ritual continuity, and intergenerational moral education. Their authority operates through everyday social practice and culturally recognized responsibilities rather than formal institutional leadership, illustrating how environmental governance is reproduced through decentralized forms of moral influence embedded within communal life.

The fourth theme, simplicity and moral restraint, demonstrates that sustainability is reinforced through everyday practices that regulate consumption, environmental interaction, and social behavior. Ecological responsibility is maintained through internalized moral discipline encouraging moderation and discouraging excessive exploitation of natural resources. Sustainability therefore emerges through routine social practice as much as through ritual obligation and cosmological belief.

Finally, the theme of lived religion and ecology illustrates how environmental preservation functions as an integral aspect of religious life rather than as a separate environmental concern. Ecological responsibility is embedded within ordinary routines, moral obligations, ritual participation, and collective social expectations. Religion therefore operates as a practical mechanism through which environmental stewardship is enacted and reproduced within everyday life.

Taken together, the themes summarized in Table 1 demonstrate that environmental governance within the Kajang community is best understood as a form of sacred ecology in which religion, cosmology, gender, morality, and environmental responsibility are mutually constitutive dimensions of collective social life. The table therefore provides an empirical foundation for the broader theoretical discussions developed in the following section concerning lived religion, ecofeminism, indigenous cosmology, and environmentally embedded governance.

## 5 Discussion

### 5.1 Sacred ecology and lived environmental governance

The findings of this study demonstrate that environmental governance within the Kajang community operates through a form of sacred ecology in which cosmological belief, ritual participation, moral discipline, and ecological responsibility are mutually embedded within everyday social life. This finding challenges approaches that understand environmental sustainability primarily through technocratic governance, state-centered environmental policy, or formal institutional regulation (Dryzek, 2021; Escobar, 2018). Rather than functioning as an externally imposed conservation strategy, environmental preservation within the Kajang community is sustained through culturally embedded systems of meaning that shape ecological behavior and collective responsibility from within the community itself.

The Kajang case illustrates that ecological sustainability is reproduced through lived religion rather than through environmental regulation alone. Environmental responsibility is enacted through ritual participation, ecological restraint, intergenerational moral transmission, and socially shared cosmological obligations that become integrated into ordinary social life. This supports and extends scholarship on lived religion, which emphasizes that religion operates not only through formal institutions or doctrinal systems but also through embodied practices, routine activities, and everyday forms of moral engagement (Ammerman, 2021; McGuire, 2008; Orsi, 2016). In the Kajang context, ecological conduct itself becomes a form of lived religious practice through which sacred meanings are continuously enacted and reproduced.

This interpretation contributes to ongoing discussions within religion-and-ecology scholarship concerning lived environmental practice, religious environmentalism, and culturally embedded sustainability (Grim and Tucker, 2014; Jenkins, 2009; Jenkins et al., 2018; Taylor, 2009, 2015; Tucker and Grim, 2001).

The present study extends these discussions by showing how environmental governance may emerge through routine social participation and internalized moral discipline rather than through explicit environmental management structures. Ecocentric values embedded in Pasang ri Kajang emphasize that nature is the center of human life, there is interdependence between humans and nature, and harmony between humans and nature must be maintained (Talib et al., 2024). Studies have documented that the Kajang community recognizes 65 plant species for various applications ranging from traditional medicine, ritual practices, to culinary uses (Thematic Research Group Hasanuddin University, 2025), demonstrating the deep botanical knowledge that underlies their conservation practices. The integration of customary governance into Nature-based Solutions (NbS) frameworks illustrates how indigenous governance systems can integrate ecological management with social justice and intergenerational equity (Ardilla et al., 2025; Ridhoh and Alfian, 2025). Sacred ecology therefore operates not only as a worldview but also as a socially effective system of ecological regulation.

The findings further suggest that cosmological belief, ritual participation, and ecological discipline should not be treated as separate dimensions of social life. Within the Kajang community, these elements function as mutually reinforcing aspects of a broader moral system that organizes relationships between humans, nature, ancestors, and collective life. Environmental responsibility acquires legitimacy because it is embedded within shared understandings of cosmological order and communal obligation. Ecological governance is therefore maintained through socially internalized commitments that shape environmental conduct at the level of everyday practice.

More broadly, the Kajang case challenges analytical distinctions that separate religion, ecology, and governance into discrete domains of inquiry. Instead, the findings suggest that environmental sustainability may be more accurately understood as a socio-religious process reproduced through ongoing interactions between cosmological meaning, ritual practice, collective participation, and moral discipline. This perspective contributes to broader debates concerning alternative forms of environmental governance by highlighting how indigenous religious systems may sustain ecological continuity through culturally grounded mechanisms of sacred ecological responsibility.

The claim of ecological sustainability is grounded in several supporting field data. First, the Ammatoa Kajang Customary Forest, covering an area of 313.99 hectares, has been officially recognized by the government and remains well-preserved to this day (ANTARA News, 2017; Ministry of Environment and Forestry, 2016). This forest is the first customary forest in Indonesia to be designated by the government, signifying recognition of the effectiveness of customary management (Nurkhalis et al., 2020).

Second, a comparative study from Forests, Trees and Agroforestry shows that the Kajang customary forest still contains old-growth forest and high-value economic species, in contrast to the state-protected forests in neighboring sub-districts, which have undergone rapid conversion into settlements (Forests, 2018). This comparison provides a strong indication that the Kajang customary governance system may be more effective in maintaining forest cover than the state protection system in the same region.

Third, this customary forest functions as a conservation area for agriculture and a source of clean water for communities in three watersheds (Raowa, Baonto, and Apparang), and possesses biodiversity potential in the form of endemic flora and fauna of South Sulawesi (Adaptation Fund, 2024; Nurkhalis et al., 2020). Fourth, the Kajang community has received an award from the Minister of Environment and Forestry for their ability to care for the forest through local wisdom (PPID KLHK, 2016).

Nevertheless, it must be acknowledged that this study did not collect independent ecological data such as longitudinal forest cover measurements, biodiversity indices, or carbon data. The claim of ecological sustainability in this study is primarily based on participant narratives, observation, and secondary data from other studies. Therefore, this claim is more appropriately understood as an indication or alignment with sustainability principles, rather than as definitive causal evidence. Further research employing a mixed-methods approach that combines qualitative and quantitative data (e.g., remote sensing, biodiversity measurement) is required to more rigorously test the relationship between customary governance systems and ecological outcomes.

### 5.2 Reframing ecofeminism through indigenous cosmology

The findings also contribute to ecofeminist scholarship by demonstrating that women’s ecological participation within the Kajang community is inseparable from cosmological belief, ritual continuity, and lived religious practice. Classical ecofeminist scholarship has long emphasized the relationship between women and ecological care within systems of domination that simultaneously exploit both women and nature (Plumwood, 1993; Shiva, 1988). At the same time, ecofeminist theory has been criticized for occasionally reproducing essentialist assumptions that portray women as naturally closer to nature than men (Arora-Jonsson, 2011; Gaard, 2011). The Kajang case provides an opportunity to reconsider these debates through an indigenous religious context in which ecological responsibility is embedded within culturally specific systems of cosmological meaning.

The findings suggest that women’s environmental participation in the Kajang community should not be understood as the expression of an inherent or universal feminine affinity with nature. Rather, women’s ecological roles emerge through socially situated processes of moral responsibility, ritual participation, customary obligations, and intergenerational transmission of cosmological knowledge, consistent with contemporary ecofeminist approaches emphasizing contextual and socially embedded forms of environmental engagement (Agarwal, 2010; Gaard, 2015).

The Kajang case therefore extends ecofeminist discussions by illustrating how women’s ecological authority may be embedded within indigenous cosmological systems and lived religious practices. Women contribute not only to environmental stewardship but also to the maintenance of the moral and symbolic foundations upon which ecological responsibility depends. Through ritual preparation, domestic ecological regulation, moral education, and intergenerational transmission of customary values, women participate in reproducing the sacred ecological order that sustains environmental governance within the community.

Importantly, the findings suggest that women’s authority is not primarily institutional. Existing discussions of religious authority frequently focus on formal leadership positions, textual interpretation, or organizational power. In contrast, women within the Kajang community exercise influence through decentralized forms of moral authority embedded within everyday practice and social relationships. Their authority emerges through their role in sustaining cosmological continuity, ritual reproduction, and ecological responsibility across generations. This finding contributes to broader sociological discussions concerning non-institutional forms of religious authority and the everyday reproduction of moral order.

Further reflection on gender relations in Kajang ecological governance reveals complexities that transcend the simple dichotomy between feminine and masculine roles. On the one hand, Kajang cosmology presents a highly feminist ethos in the ideal of environmental stewardship. The concept of anrongta which literally means “our mother” (Nurjayanti, 2023) positions nature as a maternal entity requiring protection, gentleness, and care. This ethos of stewardship is reflected in the principle of kamase-mase (simplicity), which teaches a harmonious relationship between humans and nature (Arisnawawi and Aksyar, 2026), as well as in the obligation not to take more than what is needed an ethic of care classically associated with feminist values (Plumwood, 1993; Shiva, 1988).

On the other hand, the enforcement of ecological rules within the Kajang community is carried out through highly masculine mechanisms. The highest customary leadership (Ammatoa) is held exclusively by men, and he holds the authority to impose severe physical and spiritual sanctions on violators of customary rules (Taufik et al., 2024). These sanctions such as ba’bala (whipping) with three levels of punishment (cappa ba’bala, tangnga ba’bala, and poko ba’bala) and fines reaching tens of millions of rupiah (Sartini et al., 2026) demonstrate a firm and hierarchical enforcement dimension. Even more extreme is the Attunu Panroli ritual (the burning of crowbars), used as a means of proof in cases of customary violations, where the suspect is required to hold a crowbar heated until red-hot to prove their honesty or guilt (Harsito, 2020; Sartini et al., 2026; Taufik et al., 2024). This ritual, along with the Tunu Passau sanction (the burning of incense), believed to bring supernatural calamity and even death to violators (Sartini et al., 2026), represents a form of authority and legal enforcement that is harsh a practice conventionally associated with masculinity in discourses of power and customary law.

Ironically, this masculine enforcement practice is used precisely to protect and preserve the feminist ethos of stewardship namely, the protection of nature as a maternal entity (anrongta). In other words, feminist goals (the protection and care of nature) are maintained through masculine means (strict rule enforcement, physical sanctions, and hierarchical authority). This finding reinforces the argument that ecofeminism need not be understood as biological essence or rigid gender roles (Arora-Jonsson, 2011; Gaard, 2015), but rather as a fluid and contextual social practice where feminist values can emerge in cosmology and community ethos, while enforcement practices may take highly masculine forms.

Furthermore, the existence of the Anrongta position female leaders who function as advisors to the Ammatoa and play a central role in rituals and customary decision-making (Nurjayanti, 2023) demonstrates that women’s authority in the Kajang community is not marginal. The Anrongta holds important functions in the A’borong process (customary deliberation) and even assumes temporary leadership when the Ammatoa passes away until a successor is elected (Nurjayanti, 2023). Nevertheless, there are structural limitations: in the Pasang ri Kajang, women are not permitted to hold formal leadership within the customary area (Nurfadillah et al., 2023; Puji et al., 2026), so their authority is more moral, ritual, and cultural than political-institutional. This suggests that women’s ecological authority in Kajang is not singular rather, it is a combination of decision-making power (in customary deliberations), moral influence (in intergenerational value transmission), ritual responsibility (in the preparation and execution of customary ceremonies), and domestic duties (in household resource management) (Nurjayanti, 2023).

Thus, the Kajang case challenges essentialist readings of ecofeminism and enriches our understanding of how gender, power, and ecology are intertwined in complex and non-linear ways. Ecofeminism in the Kajang context cannot be reduced to merely “women saving nature” or “nature as mother” rather, it must be understood as a dynamic social field where values of care and enforcement negotiate with one another, where moral authority and formal power complement and sometimes contradict each other, and where masculinity and femininity do not exist as separate essences but rather as co-constituting practices in the reproduction of ecological governance.

The study also demonstrates the importance of bringing indigenous cosmology into dialogue with ecofeminist theory. The religious identity of the Kajang people represents a dynamic negotiation between the indigenous Patuntung belief system and Islam, resulting in a hybrid reality known as Sallang (Malliongi et al., 2024). They identify themselves as Muslims, yet understand Islamic teachings through a local lens, believing for instance that the Qur’an consists of 40 juz, of which 10 juz were revealed in Kajang in the form of Pasang (Haq et al., 2024; Malliongi et al., 2024). Their concept of worship is encapsulated in the principle jenne talluka, sembahyang tamattappuka, meaning that maintaining honest behavior and environmental preservation is the most essential form of practicing Islamic law (Haq et al., 2024; Malliongi et al., 2024). This hybridity enables the Kajang people to maintain their cultural sovereignty while remaining integrated with global religious identity (Malliongi et al., 2024; Ridhoh and Alfian, 2025).

Much ecofeminist scholarship has been developed within secular and predominantly Western intellectual traditions. While these perspectives have generated important critiques of environmental exploitation and gender inequality, they have often paid less attention to indigenous religious systems in which environmental responsibility is organized through sacred cosmological frameworks. The Kajang case suggests that ecofeminist analysis may be enriched by greater attention to cosmological belief, ritual obligation, and lived religious practice as constitutive dimensions of ecological responsibility.

More broadly, these findings indicate that women’s ecological participation should be understood as culturally embedded and historically situated rather than universally defined. Ecological care within the Kajang community emerges through the interaction of gender, religion, cosmology, ritual practice, and communal moral expectations. This perspective contributes to contemporary ecofeminist scholarship by demonstrating how women’s environmental authority may operate through indigenous systems of sacred ecology that challenge both essentialist assumptions and narrowly institutional understandings of power.

### 5.3 Indigenous cosmology as environmental governance

Another important contribution of this study concerns the role of indigenous cosmology in sustaining environmentally embedded governance systems. The findings demonstrate that ecological regulation within the Kajang community is reproduced through cosmological understandings that connect humans, ancestors, ritual obligations, and ecological continuity within a shared moral universe. Rather than functioning merely as symbolic belief systems, cosmological understandings actively organize environmental behavior and shape collective expectations regarding appropriate relationships between humans and the natural world.

The concept of *anrongta* provides a particularly important example of how cosmological belief operates as a mechanism of environmental governance. By positioning nature as a sacred maternal entity, *anrongta* establishes a moral framework through which environmental preservation becomes a collective obligation rather than a purely technical or managerial concern. Forest protection therefore acquires legitimacy through ancestral authority, ritual obligations, and communal moral responsibility rather than through external regulatory enforcement alone.

This finding supports and extends scholarship on indigenous cosmology and sacred ecology, which argues that environmental sustainability frequently emerges through relational understandings of human–nature interaction rather than through technocratic intervention alone (Berkes, 2017; Descola, 2013; Escobar, 2018; Kohn, 2013).

Within the Kajang community, ecological responsibility is embedded within a cosmological order that links environmental conduct to broader understandings of social harmony, ancestral continuity, and moral obligation. Environmental governance therefore becomes inseparable from the cultural and religious meanings through which ecological relationships are interpreted and maintained.

The findings further suggest that indigenous cosmology functions as a socially operational system of environmental regulation. Ecological behavior is not guided primarily by formal environmental policies or bureaucratic monitoring but by internalized understandings of responsibility reinforced through ritual participation, communal expectations, and customary discipline. In this sense, cosmological belief provides both the moral justification and the social legitimacy necessary for sustaining long-term environmental stewardship.

Importantly, the Kajang case challenges dominant governance paradigms that frequently separate environmental management from cultural, religious, and cosmological systems of meaning. Much contemporary environmental governance literature continues to emphasize institutional regulation, policy frameworks, and administrative mechanisms as primary drivers of ecological sustainability. While such approaches remain important, the findings of this study demonstrate that environmental responsibility may also be sustained through culturally embedded systems of sacred ecological obligation that operate through everyday social life.

This perspective contributes to broader discussions concerning decolonial and community-based approaches to sustainability by highlighting the governance capacities of indigenous knowledge systems and cosmological traditions (Escobar, 2018). Indigenous environmental governance should therefore not be reduced to traditional ecological knowledge alone. Rather, it should be understood as a dynamic socio-religious system in which cosmology, ritual practice, moral discipline, and collective participation function together to regulate environmental behavior and sustain ecological continuity.

At the same time, the findings indicate that indigenous cosmological systems continue to encounter pressures arising from modernization, economic transformation, external governance interventions, and changing generational aspirations. The challenges of modernity and technological penetration are currently beginning to influence the mindset of the younger Kajang generation, who are starting to question the relevance of traditional lifestyles (Nurfatimah et al., 2025; Ridhoh and Alfian, 2025). To protect the sovereignty of their territory, the Bulukumba Regency Government has enacted Regional Regulation No. 9 of 2015 as a formal legal basis for the recognition of customary rights and customary forests (Dandi and Syahribulan, 2025; Nurfatimah et al., 2025). Although the formal recognition of Ammatoa Kajang Customary Forest by the state provides strong legal protection, the sustainability of this system remains dependent on the community’s internal consistency in transmitting Pasang values across generations (Nurfatimah et al., 2025; Ridhoh and Alfian, 2025). The comprehensive social order in Kajang demonstrates that spirituality-based local wisdom can serve as a more effective conservation instrument than mere administrative regulation (Ardilla et al., 2025; Ridhoh and Alfian, 2025).

The effectiveness of cosmologically embedded environmental governance therefore depends upon the continued reproduction of sacred ecological values across generations. Consequently, environmental sustainability within the Kajang community should not be understood as a static cultural condition but as an ongoing process of negotiation, adaptation, and moral renewal.

More broadly, the Kajang case demonstrates that indigenous cosmology may function not only as a framework of environmental meaning but also as a practical mechanism of governance. By moral obligation, ritual continuity, collective participation, and sacred ecological responsibility, cosmological systems provide alternative pathways through which environmental sustainability can be organized and maintained. This finding contributes to contemporary debates concerning plural approaches to environmental governance and highlights the continuing relevance of indigenous religious traditions in addressing ecological challenges within an increasingly uncertain environmental future.

### 5.4 Everyday moral discipline and sustainable social life

The findings further demonstrate that ecological sustainability within the Kajang community is sustained through everyday forms of moral discipline that regulate patterns of consumption, environmental interaction, and social conduct. Practices of simplicity, restraint, moderation, and collective responsibility are not experienced merely as customary expectations but as morally meaningful orientations that shape how individuals understand their relationship with nature and with the broader social community.

This finding extends discussions concerning lived religion by illustrating how environmental responsibility becomes embedded within ordinary routines and habitual forms of conduct. Religion, in this context, operates not only through ritual events or explicit theological reflection but also through everyday practices that shape moral dispositions and guide environmental behavior (Ammerman, 2021; McGuire, 2008; Orsi, 2016). Ecological restraint therefore functions as an embodied expression of sacred ecological ethics reproduced through daily participation in communal life.

The Kajang case suggests that sustainability is maintained not solely through ecological knowledge or formal regulation but through the cultivation of moral orientations that encourage moderation and discourage excessive exploitation of nature. Participants consistently associated environmental degradation with greed, excess, and the disruption of reciprocal relationships between humans and the natural world. Ecological sustainability is therefore linked to the regulation of desire and the maintenance of moral balance rather than simply to technical forms of environmental management.

This interpretation resonates with broader scholarship on sacred ecology and indigenous environmental ethics, which emphasizes the importance of relational responsibility, reciprocity, and moral obligation in sustaining ecological continuity (Berkes, 2017; Grim and Tucker, 2014; Taylor, 2009). Within the Kajang community, environmental restraint is not merely a practical strategy for resource conservation but part of a larger moral system that links human well-being, communal harmony, and ecological balance. Sustainability emerges because ecological responsibility is internalized as a moral commitment rather than imposed as an external requirement.

The findings further indicate that everyday moral discipline contributes to the reproduction of environmentally sustainable social life by connecting ecological behavior to collective identity and communal belonging. Environmental conduct is evaluated not only in relation to its ecological consequences but also in terms of its moral significance for maintaining social harmony and honoring ancestral teachings. This reinforces the idea that environmental sustainability is fundamentally embedded within broader systems of cultural meaning and social responsibility.

At the same time, the findings reveal that these systems of moral discipline are increasingly negotiated within changing social and economic contexts. Expanding market relations, consumer culture, formal education, and broader processes of modernization create new opportunities as well as new challenges for the reproduction of customary ecological values. Sustainability within the Kajang community should therefore be understood not as a static cultural achievement but as an ongoing social process that requires continuous moral reinforcement and collective participation.

#### 5.4.1 Negotiating identity and situational compliance among the younger generation

Contact with the outside world has created a space for negotiation among the younger generation of Kajang, particularly those residing in the Outer Kajang area (Ipantarang Embayya) a customary region where customary rules are not fully enforced and community members are permitted to adopt external value systems. In this area, access to formal education, communication technology, and economic interaction with outsiders is more intensive compared to Inner Kajang (Ilalang Embayya) (Alvira et al., 2024; Arisnawawi, 2022). This means that engagement with the outer Kajang community constitutes a primary channel for negotiation processes, especially among the youth.

The younger generation of Kajang living in Outer Kajang appears to be gradually affirming the values and lifestyles of Bulukumba in general. This is supported by their accessibility to formal education, social media, and economic interaction with the broader society. Although there is not yet visible resistance to customary values, this access opens up possibilities for them to encounter new things from the outside world (Arianti et al., 2025).

Economic relations with outsiders are most tangible in the dynamics of value negotiation among the younger generation. Economic activities such as trading forest and agricultural products, as well as labor migration to larger cities like Makassar, create an inevitable exchange of information and experience. Young people who work outside the community or interact with outside traders inevitably experience realities of a different society. This process may subtly and gradually encourage acculturation.

Formal education serves as one of the primary channels for the entry of external influences into the Kajang community (Arianti et al., 2025). Young Kajang people who attend school outside the customary area acquire formal (scientific) knowledge and new ways of thinking that differ from traditional understandings. However, formal education does not necessarily erase cultural identity; rather, it can add another layer of knowledge that may be integrated with existing customary values.

It is important to note that the negotiation processes occurring among the younger generation of Kajang have not yet demonstrated resistance to the customary value system as a whole. Instead, what is observable is a gradual process of adaptation in which the younger generation attempts to remain compliant with values, even though they cannot entirely distance themselves from the outside world. Thus, they face a situation where their cultural identity confronts the demands and opportunities of the modern world. Consequently, the future will reveal the extent to which this negotiation continues and how the younger generation of Kajang will define their identity amid the ongoing currents of change.

#### 5.4.2 Challenges from government and Forest exploitation for capitalist interests

Although internal dynamics contain potential for weakening, particularly among the younger generation, the greatest threat to the sustainability of Kajang customary forest governance comes from government policies. This is because the government’s position is more open to capitalist expansion, which often disregards the rights of indigenous communities. Although the Kajang customary forest has received recognition through Bulukumba Regency Regional Regulation Number 9 of 2015, conflicts with external parties remain unavoidable one of which is the conflict with PT London Sumatera (Lonsum).

PT London Sumatera (Lonsum) is a private plantation company that claims and manages land within the Kajang customary area. The South Sulawesi Regional Legislative Council followed up on the aspirations of the Bulukumba community regarding the alleged seizure of customary land by PT Lonsum by visiting the Directorate General Office of the Ministry of Agrarian Affairs and Spatial Planning/National Land Agency in Jakarta (ANTARA News, 2024). There are three main clusters in resolving this issue: first, there is overlap between the Cultivation Rights (Hak Guna Usaha/HGU) and the territory of the Kajang customary law community; second, the community’s claim over land not included in the HGU despite a Supreme Court ruling cannot yet be implemented due to differing perceptions between the community and PT Lonsum; and third, certificates have already been issued within the HGU area.

Within the legal domain, the Bulukumba District Court rejected all land dispute lawsuits filed by nine residents against the Kajang customary leader, Puto Palasa, in accordance with the verdict in case Number 9/Pdt. G/2025/PN Blk (ANTARA News, 2026; Dandapala, 2026). However, these claims were refuted by the defendant, the customary leader, who affirmed that the land in question is part of the Kajang customary territory. Referring to Bulukumba Regency Regional Regulation Number 9 of 2015, the location of the disputed object is known to be within the customary forest area under the authority and legal protection of the Ammatoa Kajang customary law (ANTARA News, 2026).

In addition to conflicts with plantation companies, threats also come from infrastructure development plans that disregard the status of customary areas. The Bulukumba Regency Government has decided that land within PT Lonsum or the Ammatoa Kajang customary area will be used as a motocross arena. This plan demonstrates how capitalist and development interests often disregard the legally recognized status of customary areas. This case also reflects that formal recognition through Regional Regulations and the Minister of Environment and Forestry Decree is not sufficient to protect customary areas from intervention by local governments oriented toward economic and tourism interests.

These conflicts manifest in the form of indigenous peoples’ movements and limited solidarity from other parties. The Ammatoa, as the highest customary leader, emphasizes that customary institutions and the government must cooperate in preserving the forest. However, the reality on the ground shows that this synergy remains dynamic, given the ongoing tensions between competing interests and efforts to convert customary land for purposes outside the community.

Coordination constraints in preserving the Ammatoa Kajang customary forest are also currently caused by ineffective communication due to sectoral ego among institutions, and a lack of communication among Kajang community actors to build greater solidarity. This further complicates efforts to protect the customary forest from external threats.

More broadly, the Kajang case contributes to contemporary sustainability debates by demonstrating that environmental responsibility may be reproduced through culturally embedded moral systems operating within everyday life. In contrast to approaches that rely primarily on policy intervention or institutional regulation, the findings highlight the importance of ordinary social practices, moral discipline, and sacred ecological ethics in sustaining long-term environmental stewardship. This perspective underscores the continuing relevance of indigenous religious traditions as sources of ecological responsibility and sustainable social life in an era of intensifying environmental uncertainty.

### 5.5 Theoretical contributions to the sociology of religion

This study contributes to the sociology of religion in several important ways by demonstrating how environmental sustainability may be reproduced through everyday religious practice, indigenous cosmology, and decentralized forms of moral authority. More specifically, the findings extend scholarship on lived religion, religious authority, and the relationship between religion and ecological governance by showing how environmental responsibility becomes embedded within ordinary social life.

First, the study expands the concept of lived religion by demonstrating that ecological practices themselves may function as forms of religious action. Existing scholarship on lived religion has emphasized that religion operates not only through institutional structures and formal doctrines but also through embodied practices, routine activities, and everyday moral conduct (Ammerman, 2021; McGuire, 2008; Orsi, 2016). The findings of this study support these arguments while extending them into the domain of environmental governance. Within the Kajang community, environmental preservation is not experienced as a separate environmental concern but as an integral aspect of religious and moral life. Ecological responsibility is reproduced through ritual participation, customary discipline, intergenerational transmission, and everyday environmental conduct. This suggests that environmental practices themselves can be understood as expressions of lived religion through which sacred meanings are enacted and sustained.

Second, the study contributes to sociological discussions concerning religious authority by highlighting the significance of decentralized and non-institutional forms of moral influence. Much scholarship on religious authority has focused on formal leadership positions, doctrinal interpretation, institutional structures, and recognized religious specialists. The Kajang case demonstrates that religious authority may also operate through ordinary social relationships and everyday practices. Women, in particular, contribute to the reproduction of ecological responsibility through moral education, ritual continuity, domestic ecological regulation, and the transmission of cosmological values. Their authority is exercised not through bureaucratic office or formal leadership but through socially recognized responsibilities that sustain communal moral order. This finding broadens sociological understandings of how religious authority is produced, maintained, and reproduced within everyday social life.

Third, the study contributes to scholarship on religion and ecology by illustrating how indigenous cosmological systems may function as practical mechanisms of environmental governance. Existing studies have often examined religion as a source of environmental ethics, ecological values, or symbolic understandings of nature (Grim, 2001; Grim and Tucker, 2014; Taylor, 2009). The findings of this study extend these discussions by showing that indigenous religious systems may also organize concrete forms of environmental regulation through ritual participation, moral discipline, collective obligation, and customary ecological responsibility. Environmental governance therefore emerges not only through formal institutional arrangements but also through culturally embedded religious systems that shape environmental behavior from within everyday communal life.

Fourth, the findings challenge analytical distinctions that separate religion, ecology, governance, and morality into discrete sociological domains. Within the Kajang community, these dimensions operate as mutually constitutive aspects of a broader socio-religious system. Cosmological belief, ritual practice, ecological responsibility, gendered participation, and communal moral obligations are continuously reproduced through everyday interaction and collective participation. This perspective suggests that environmental sustainability may be more productively understood as a socio-religious process rather than solely as an environmental or governance outcome.

Finally, the study contributes to ongoing efforts within the sociology of religion to engage more seriously with indigenous religious traditions and non-Western forms of religious life. Much sociological scholarship continues to be shaped by conceptual frameworks derived primarily from institutionalized religious traditions and modern organizational forms. The Kajang case demonstrates the analytical value of examining indigenous cosmological systems in which religion is embedded within ecological relations, customary obligations, and collective moral practice. By bringing lived religion, ecofeminism, sacred ecology, and indigenous cosmology into a single analytical framework, this study highlights the importance of culturally grounded religious systems for understanding contemporary questions concerning environmental sustainability, moral regulation, and collective social life.

Taken together, these contributions suggest that religion should be understood not only as a system of belief or institutional organization but also as a socially embedded process through which ecological responsibility, moral authority, and environmental governance are reproduced in everyday life. The Kajang case therefore expands sociological understandings of religion by demonstrating how sacred ecological systems operate as practical mechanisms of environmental continuity, communal regulation, and sustainable social existence.

Table 2 demonstrates that environmental governance within the Kajang community operates through the intersection of lived religion, sacred ecology, ecofeminism, and indigenous cosmology as mutually embedded dimensions of collective social life. Ecological sustainability is reproduced not primarily through formal institutional regulation but through ritual participation, communal moral discipline, gendered ecological responsibility, and cosmologically grounded environmental ethics.

**Table 2:** Integration of theoretical perspectives and empirical findings.

Theoretical perspective	Core concept	Empirical findings in the Kajang community	Theoretical contribution
Lived Religion	Religion as embodied everyday practice	Ecological preservation enacted through ritual participation and moral discipline	Expands lived religion into environmental governance
Ecofeminism	Gendered ecological responsibility	Women function as custodians of sacred ecological morality	Reframes ecofeminism within indigenous cosmology
Indigenous Cosmology	Nature as sacred relational order	Forest protection linked to ancestral cosmology and ritual obligation	Demonstrates cosmology as environmental governance
Sacred Ecology	Ecology embedded in religious morality	Environmental sustainability reproduced through collective sacred ethics	Extends sacred ecology into lived governance systems
Sociology of Religion	Moral regulation and social participation	Ecological discipline sustained through communal moral obligation	Religion operates as socially embedded sustainability mechanism

The findings further reveal that women occupy central positions within this governance system through intergenerational moral transmission, ritual continuity, and everyday ecological stewardship. Taken together, the findings demonstrate that sacred ecology functions as a culturally embedded mechanism of indigenous environmental governance sustained through lived religious practice, socially recognized moral authority, and collective cosmological responsibility.

## 6 Conclusion

This study has examined how lived religion, indigenous cosmology, and gendered moral participation shape environmentally embedded governance within the Kajang indigenous community of South Sulawesi, Indonesia. The findings demonstrate that ecological sustainability within the Kajang community is not maintained primarily through formal institutional regulation or technocratic environmental policy but through sacred ecological ethics embedded within everyday social life. Environmental responsibility is reproduced through cosmological belief, ritual participation, moral discipline, ecological restraint, and collectively shared understandings of reciprocal obligations between humans and nature.

This study has examined how lived religion, indigenous cosmology, and gendered moral participation shape environmental governance within the Kajang indigenous community of South Sulawesi, Indonesia. The findings demonstrate that ecological sustainability is maintained not through formal institutional regulation but through sacred ecological ethics embedded within everyday social life. Environmental responsibility is reproduced through cosmological belief, ritual participation, moral discipline, ecological restraint, and collectively shared understandings of reciprocal obligations between humans and nature.

Central to this system is the cosmological concept of anrongta, which positions nature as a sacred maternal entity requiring protection and communal stewardship. This cosmology is operationalized through a structured forest zoning system (Borong Karama, Borong Batasayya, Borong Luarayya) reinforced by customary sanctions (ba’bala) and ritual practices including Andingingi and Akbattasa Jera. Through this framework, environmental preservation acquires religious significance and becomes integrated into broader systems of moral obligation and collective responsibility.

Women occupy central positions through the institutionalized role of Anrongta, functioning as advisor to the Ammatoa and participant in customary deliberations. Their authority operates through decentralized forms of moral influence rather than formal leadership. Significantly, the Kajang case reveals a complex gender dynamic in which feminist goals of environmental care are enforced through masculine mechanisms, including strict rule enforcement and physical sanctions, demonstrating that ecofeminism in this context transcends essentialist binaries.

The study advances three theoretical contributions: it extends lived religion scholarship by demonstrating ecological practices as forms of religious action; it shows how religious authority may emerge through decentralized moral influence; and it demonstrates that indigenous cosmological systems function as practical mechanisms of environmental governance, supported by secondary ecological data including formal recognition of the 313.99-hectare customary forest.

The Kajang case highlights the continuing relevance of indigenous religious traditions in contemporary sustainability debates. At a time when ecological crises expose the limitations of technocratic approaches, indigenous cosmological systems provide insights into how environmental responsibility may be sustained through culturally grounded moral commitments and collective participation.

While this study focuses on a single community and acknowledges the absence of independent ecological data, the findings demonstrate that sacred ecology, lived religion, indigenous cosmology, and gendered moral authority remain significant sociological forces shaping environmental sustainability within contemporary social life.

## Data Availability

The datasets presented in this article are not readily available because the study’s findings are constrained by a 2-month data collection window, limiting temporal depth, and seasonal insights. Requests to access the datasets should be directed to najamuddin@unm.ac.id.
